# Supplementary material for: A dynamic, spatially periodic, micro‐pattern of HES5 underlies neurogenesis in the mouse spinal cord
Source: Mol Syst Biol. 2021 May 25;17(5):e9902. doi: 10.15252/msb.20209902 (PMC8144840; doi:10.15252/msb.20209902)
Supplement: Supplementary file 2 — Expanded View Figures PDF [file MSB-17-e9902-s006.pdf]

Expanded View Figures

Figure EV1. Venus::HES5 expression in single progenitor cells located in the ventral domain of spinal cord. Related to Fig 1.

A Transverse slice of live Venus::HES5 homozygous knock-in mouse spinal cord E10.5 *ex vivo*, Draq5 live nuclear stain and brightfield image. Scale bar 50 μm.

B Immunofluorescence of E10.5 Venus::HES5 transverse slice of spinal cord *ex vivo*. SOX2+ progenitors and endogenous Venus::HES5 signal. Scale bar 40 μm.

C Viridis look-up table applied to mean nuclear Draq5 intensity corresponding to transverse slice in Fig 1C.

D Total number of cells per microcluster detected in Venus::HES5 segmented images versus synthetically generated randomised images (see Materials and Methods); dots indicate individual clusters detected in 14 segmented images with 20x repeats per slice of Rnd1 (random permutation of real intensity observations) and Rnd2 (randomised intensities with same mean and SD); bars indicate mean and SD; Kruskal–Wallis *t*-test with Dunn's multiple comparison, significance ****$P < 0.0001$.

E Pearson correlation coefficient of mean nuclear Venus::HES5 intensity in relationship to distance for (left panel) E9.5 and (right panel) E11.5 Venus::HES5 spinal cord *ex vivo* slices; red dots indicate average Venus::HES5 correlation per slice of three slices from three experiments with corresponding red line indicating one phase decay fit. Black line denotes 95% confidence levels. Grey dots indicate correlations from randomisations of intensities analysed in the same way.

F Transverse slice of live (left panel) Venus::HES5 homozygous knock-in mouse spinal cord E10.5 *ex vivo* showing (middle panel) segmentation of Draq5 and (right panel) mask applied to Venus::HES5 channel. Images correspond to slice shown in Fig 1G. Scale bar 30 μm, D—dorsal, V—ventral.

G, H Left panels—Viridis look-up table applied to mean nuclear Venus::HES5 intensity in E9.5 and E11.5 slices, respectively, after radial gradient removal. Right panels—Pearson correlation coefficient of mean nuclear Venus::HES5 intensity in relationship to distance for E9.5 and E11.5 Venus::HES5 spinal cord *ex vivo* slices, respectively. Red dots indicate average Venus::HES5 correlation per slice of three slices from three experiments with corresponding red line indicating one phase decay fit. Black line denotes 95% confidence levels.

 © 2021 The Authors

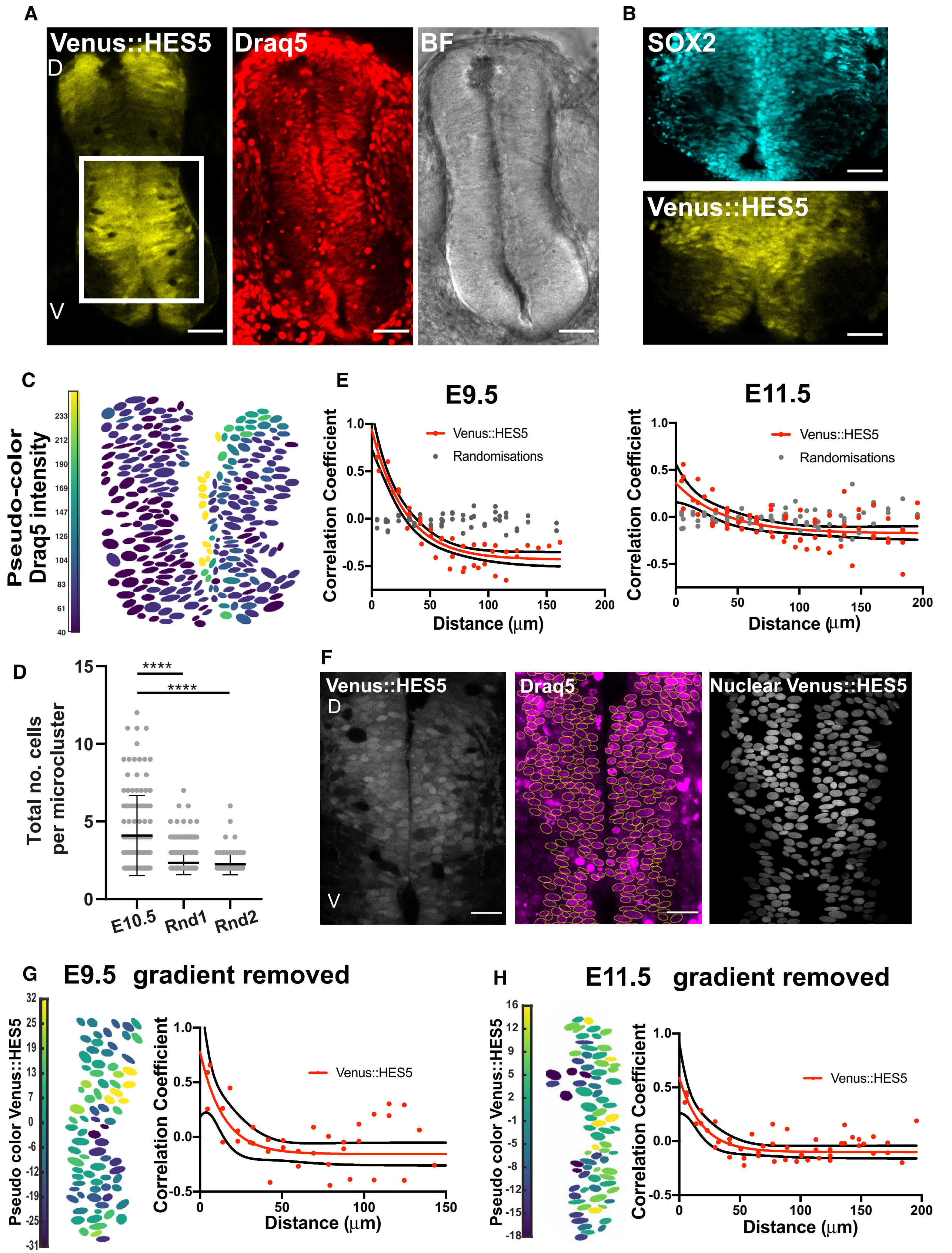
A
Venus::HES5
D
V
Draq5
BF
B
SOX2
Venus::HES5
C
Pseudo-color Draq5 intensity
E
E9.5
E11.5
Correlation Coefficient
Distance (μm)
Venus::HES5
Randomisations
D
Total no. cells per microcluster
****
****
E10.5
Rnd1
Rnd2
F
Venus::HES5
Draq5
Nuclear Venus::HES5
G
E9.5 gradient removed
Pseudo color Venus::HES5
H
E11.5 gradient removed

Figure EV1.

Figure EV2. Draq5 and Venus::HES5 spatial periodicity in live spinal cord tissue slices. Related to Fig 2.

A Transverse slice of live Venus::HES5 (left panel) homozygous knock-in mouse spinal cord E10.5 *ex vivo*, Draq5 live nuclear stain (right panel); rectangle shows region for spatial profile.

B Viridis look-up table applied to mean nuclear Venus::HES5 intensity in the same slice shown in (A), rectangle shows the same region of interest for spatial profile.

C Venus::HES5 intensity spatial profile (black) from the yellow box in (a—non-segmented data), and trend line in blue (fitted polynomial order 6).

D Detrended spatial profile of Venus::HES5 (grey) and Draq5 (red) nuclear stain from region delineated in (A); arrows show regions of Venus::HES5 low and Draq5 high indicating low Venus::HES5 areas are not nuclei free.

E Distribution of peak to peak distance in auto-correlation plots of Draq5 spatial profile; this is a measure of inter-nuclear distance in Draq5 profile along dorsal–ventral axis of spinal cord; data points represent all peak to peak distances from nine slices, six experiments; mean 9.3 ± 0.42 μm (95% confidence limits).

F Inter-cluster distance in the dorsoventral (DV) axis computed from multiple microclusters detected in E10.5 spinal cord segmented images using an automated approach (see Materials and Methods); dots indicate individual observations collected from 14 images; bars indicate mean and SD.

G Spatial periodicity detected by Lomb–Scargle periodogram in apical, medium and basal regions (10, 30 and 60 μm from ventricle, respectively); dots indicate mean periodicity from at least three z-sections and both left and right sides of ventricle analysed in six experiments; lines indicate mean and SD per experiment; Kruskal–Wallis test not significant, $P = 0.3137$.

H Longitudinal cryosection of neural tube in E10.5 Venus::HES5 embryos. A—anterior, P—posterior. Scale bar 60 μm.

I Representative detrended spatial profile of Venus::HES5 from neural tube in anterior–posterior (A-P) direction.

J Representative auto-correlation of Venus::HES5 spatial profile in A-P direction. Multiple significant peaks indicate spatial periodicity in A-P direction. Significant peaks (red triangle) lie outside black dotted lines indicating 95% significance based on bootstrap approach (see Materials and Methods) and non-significant peaks (black triangles).

K Pearson correlation coefficient of detrended Venus::HES5 spatial profile between subsequent z-sections of transverse E10.5 spinal cord tissue slices at known distances (i.e. correlations in A-P direction); untreated slices—dots show 18 pairs of z-sections from six experiments; DMSO-treated slices—dots show nine pairs from three experiments.

L (Left panel) Representative spatiotemporal plot of the detrended Venus::HES5 pattern along ventral–dorsal direction (0 to 200 μm) obtained by averaging kymograph data in the same region over 2-h time intervals; (Right panel) Representative phase map of spatially periodic Venus::HES5 intensity obtained using the Hilbert transform (see Materials and Methods) from data shown in the left panel; markers indicate areas underdoing a high-to-low* and low-to-high** transition.

© 2021 The Authors

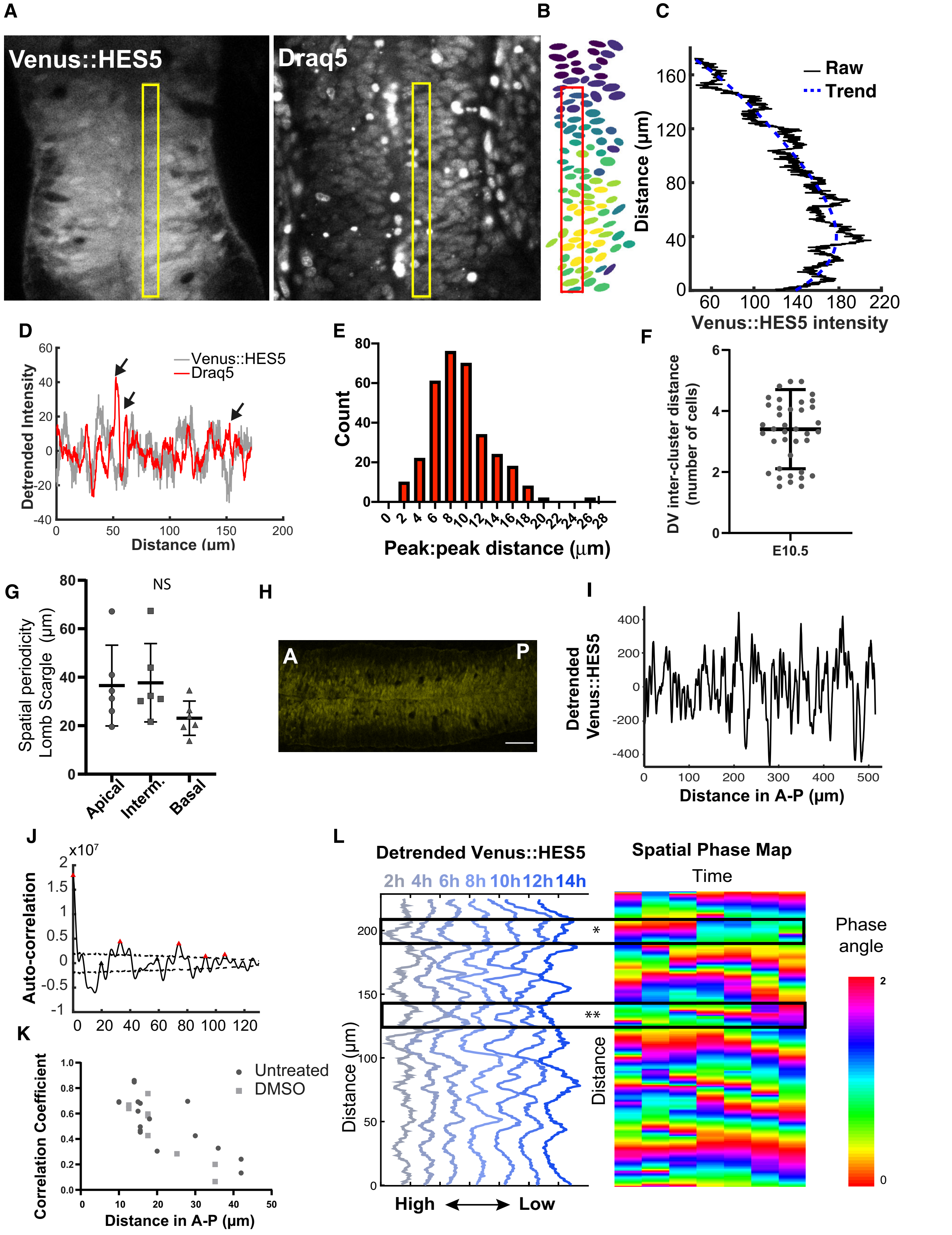
A
Venus::HES5
Draq5
B
C
Raw
Trend
Distance (µm)
Venus::HES5 intensity
D
Venus::HES5
Draq5
Detrended Intensity
Distance (µm)
E
Count
Peak:peak distance (µm)
F
DV inter-cluster distance (number of cells)
E10.5
G
NS
Spatial periodicity Lomb Scargle (µm)
Apical
Interm.
Basal
H
A
P
I
Detrended Venus::HES5
Distance in A-P (µm)
J
x10^7
Auto-correlation
K
Untreated
DMSO
Correlation Coefficient
Distance in A-P (µm)
L
Detrended Venus::HES5
2h 4h 6h 8h 10h 12h 14h
Spatial Phase Map
Time
Phase angle
Distance (µm)
Distance
High
Low

Figure EV2.

Figure EV3. Changes in Venus::HES5 spatiotemporal expression pattern in live slice cultures treated with Notch inhibitor DBZ. Related to Fig 4.

A Representative examples of the detrended Venus::HES5 signal observed along ventral–dorsal direction in DMSO and DBZ conditions; Venus::HES5 intensity data obtained by averaging kymographs over 2-h intervals; panels represent individual slice cultures (2 per condition) in addition to examples in Fig 4B.

B Spatial phase maps obtained from detrended Venus::HES5 signal in DMSO and DBZ conditions; panels correspond to Venus::HES5 intensity traces shown in (A).

C Auto-correlation analysis of detrended apical Venus::HES5 spatial profile in DMSO (control) and DBZ (2 μM) treated E10.5 Venus::HES5 spinal cord slices; panels show auto-correlation of detrended Venus::HES5 signal averaged from 0–2 h of timelapse video (top panels) and corresponding auto-correlation functions in the same slices averaged in 2 h windows for 10 h; we observed a decrease in amplitude of auto-correlation peaks over time in DBZ-treated slices.

D Peak to peak distance in auto-correlation from spatial data shown in Fig 4H. Lines show median per experiment from DMSO ($n = 3$ experiments) and DBZ ($n = 4$ experiments); 1-tailed t-test not significant $P = 0.0526$.

E Nuclear density represented by the % area covered by nuclei in DMSO and DBZ treated *ex vivo* E10.5 spinal cord slice cultures dots indicate multiple z-stacks from DBZ (5 slices) and DMSO (4 slices); bars indicate mean and SD per condition; 2-tailed t-test ***$P = 0.0004$.

© 2021 The Authors

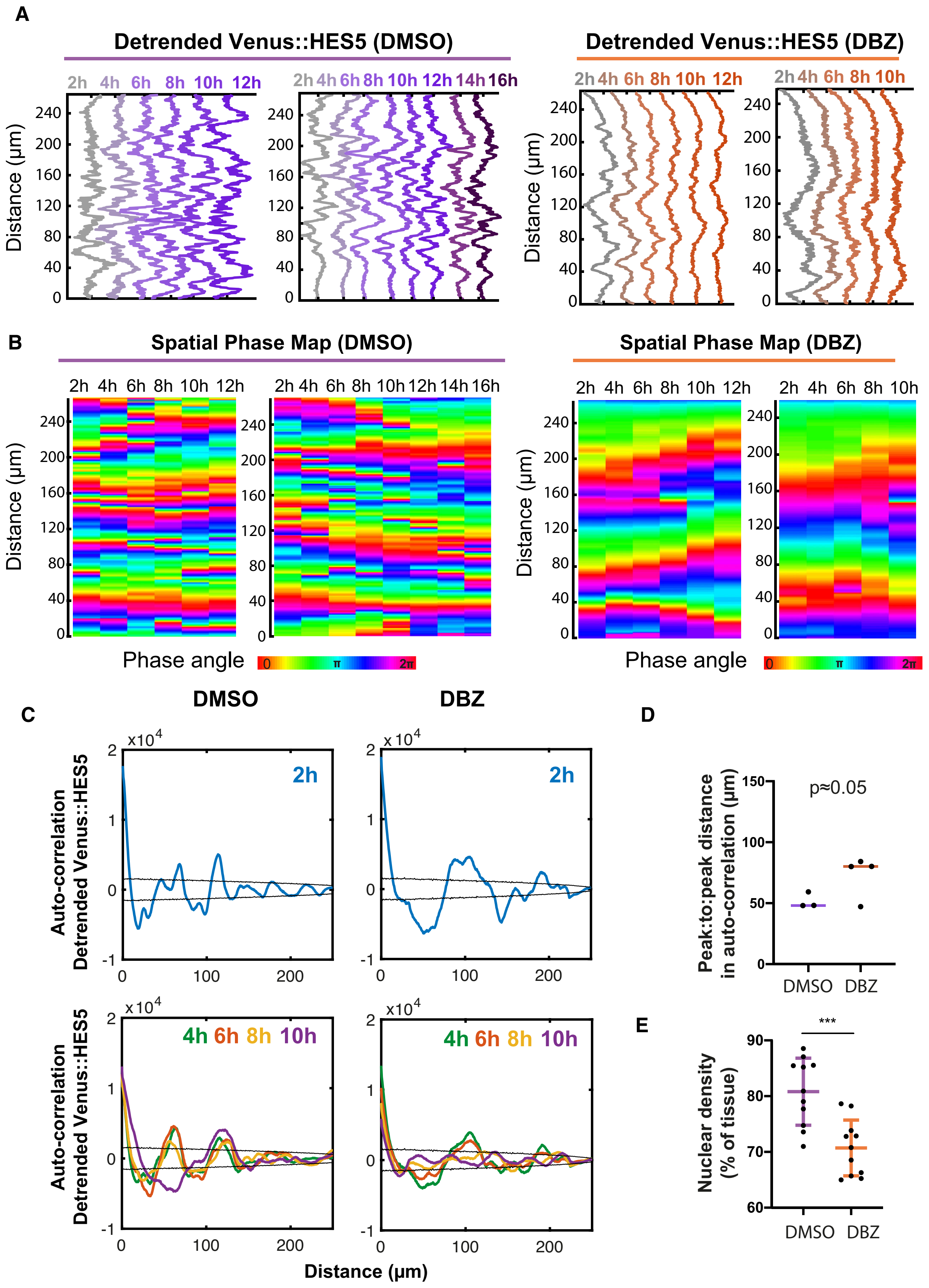
A
Detrended Venus::HES5 (DMSO)
Detrended Venus::HES5 (DBZ)
2h 4h 6h 8h 10h 12h
2h 4h 6h 8h 10h 12h 14h 16h
2h 4h 6h 8h 10h 12h
2h 4h 6h 8h 10h
Distance (µm)
B
Spatial Phase Map (DMSO)
Spatial Phase Map (DBZ)
Phase angle
0 π 2π
C
DMSO
DBZ
Auto-correlation Detrended Venus::HES5
x10^4
2h
4h 6h 8h 10h
Distance (µm)
D
Peak:to:peak distance in auto-correlation (µm)
p≈0.05
DMSO DBZ
E
Nuclear density (% of tissue)
***
DMSO DBZ

Figure EV3.

Figure EV4. HES5 spatiotemporal dynamics correlate with rate of differentiation.

A Diagram of Notch–Delta inter-cellular communication and HES5 interactions. Our model takes into account that HES5 negatively regulates its own mRNA production (grey highlighted area), downstream proneural genes and Delta; the direct and/or indirect (via proneural genes) repression of Delta by HES5 is expressed mathematically through the means of an inter-cellular Hill function (see Materials and Methods).

B Transverse cryosection of E10.5 Venus::HES5 spinal cord. Venus::HES5 endogenous signal, OLIG2: motorneuron progenitor marker, NGN2: early marker of neuronal commitment, DAPI; scale bar 25 μm.

C Spatial expression profile of Venus::HES5, NGN2 and OLIG2 from the same tissue to help delineate motorneuron (OLIG2+) vs interneuron (OLIG2-) domains.

D Spatial profile of Venus::HES5 intensity (black) generated by averaging 2.5 h of kymograph data; 0 distance represents ventral end of kymograph; blue dotted line is trend in Venus::HES5 data across the domain determined by polynomial fit order 6; domain boundary between motorneuron progenitors (MN) and interneuron progenitors (IN) marked with red dashed line.

E Detrended spatial profile of Venus::HES5 corresponding to (C) in motorneuron progenitors (red:MN) and interneuron progenitors (blue:IN).

F Auto-correlation plot of detrended Venus::HES5 spatial profile in MN and IN progenitors; black lines show confidence limits for peak significance based on bootstrap approach on detrended Venus::HES5 intensity profile (see Materials and Methods); red triangle—significant peak, black triangle—non-significant peak; multiple significant peaks in auto-correlation shows periodicity in spatial profile of Venus::HES5 intensity.

G Spatial periodicity of Venus::HES5 in motorneuron vs interneuron domain measured with the Lomb–Scargle periodogram; top 2 significant peaks were used to calculate spatial period from 2 to 3 z-sections per experiment, left and right side of ventricle analysed separately and six experiments; bars indicate mean with SD; Mann–Whitney test with two-tail significance for ****$P < 0.0001$.

H Fold-change in Venus::HES5 spatial pattern between hi-low regions in IN domain relative to MN domain. Data points represent mean per experiment. Lines shows mean and SD of six experiments. 2-tailed Mann–Whitney test **$P = 0.0022$.

© 2021 The Authors

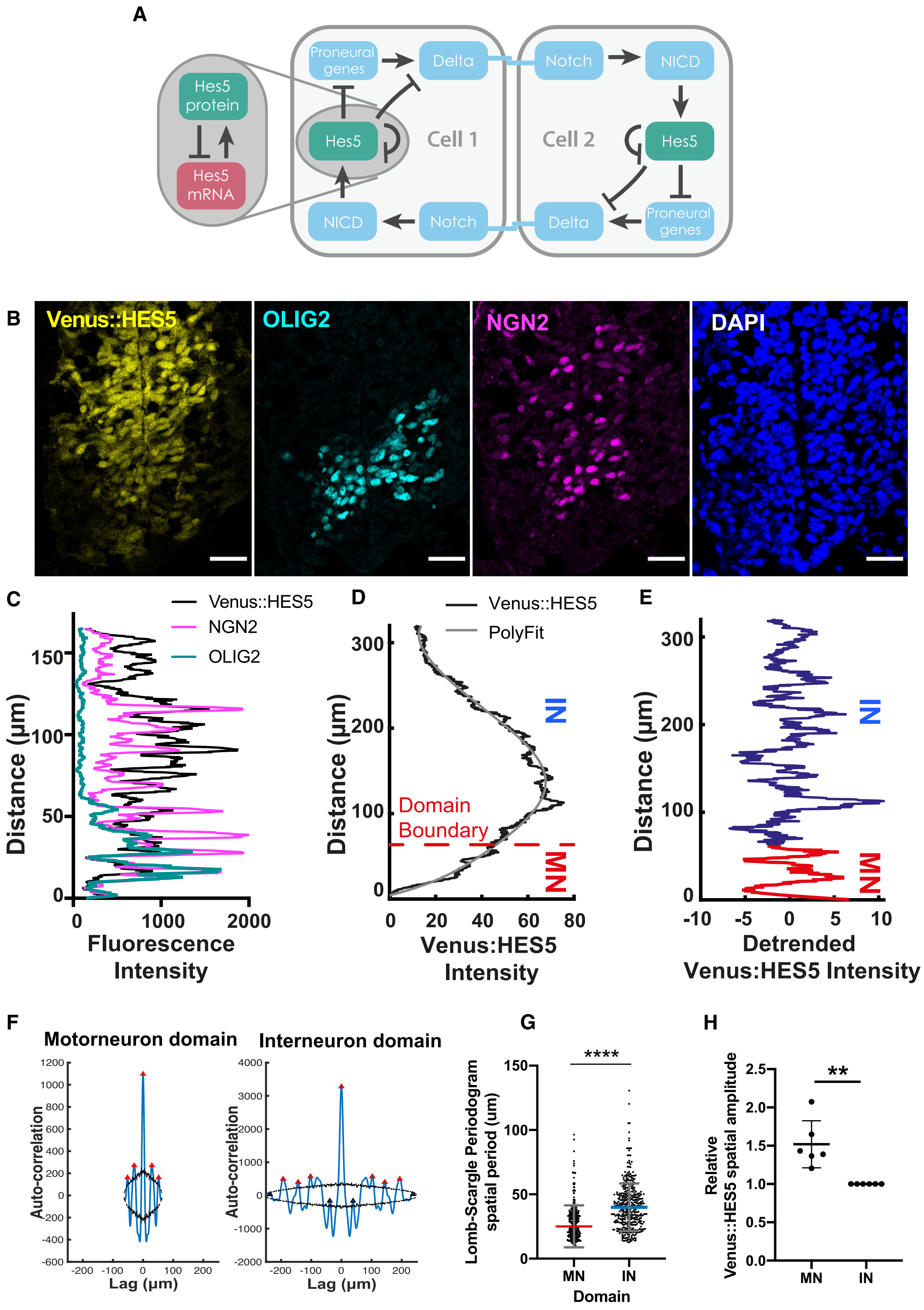
A
Hes5 protein
Hes5 mRNA
Proneural genes
Delta
Hes5
Cell 1
NICD
Notch
Notch
NICD
Cell 2
Hes5
Delta
Proneural genes
B
Venus::HES5
OLIG2
NGN2
DAPI
C
Venus::HES5
NGN2
OLIG2
Distance (µm)
Fluorescence Intensity
D
Venus::HES5
PolyFit
Distance (µm)
Domain Boundary
IN
MN
Venus:HES5 Intensity
E
Distance (µm)
IN
MN
Detrended Venus:HES5 Intensity
F
Motorneuron domain
Interneuron domain
Auto-correlation
Lag (µm)
G
Lomb-Scargle Periodogram spatial period (um)
****
MN
IN
Domain
H
Relative Venus::HES5 spatial amplitude
**
MN
IN

Figure EV4.

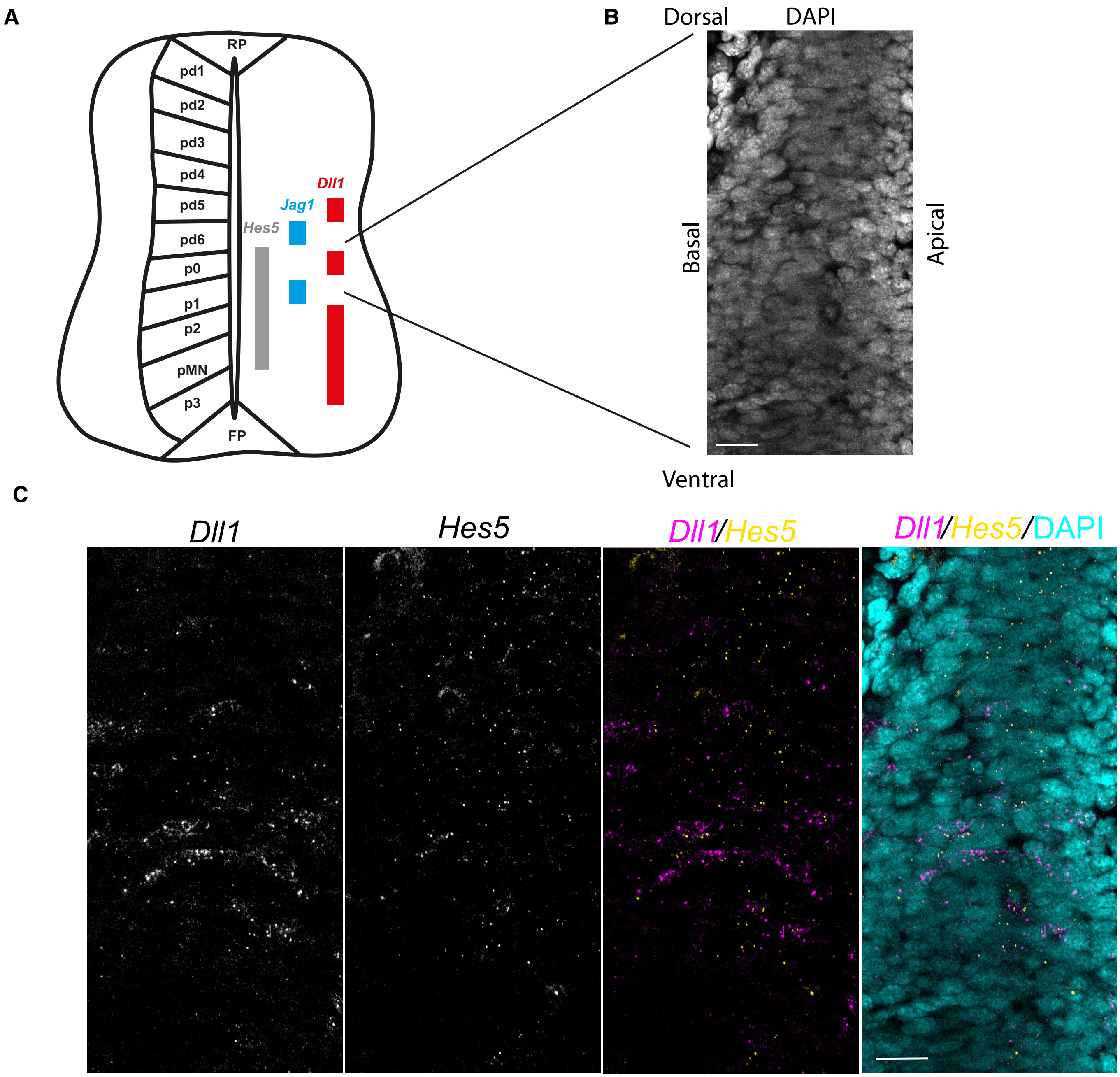

Figure EV5. Exploration of HES5 spatiotemporal dynamics in a multicellular environment.

A Schematic of *Hes5*, *Dll1* and *Jag1* expression in progenitor domains along D-V axis of E10.5 mouse spinal cord compiled based on data from Manning *et al* (2019) and Marklund *et al* (2010).

B DAPI nuclear stain of interneuron region in E10.5 mouse; lines indicate spatial localisation in tissue. Scale bar 20 μm.

C Single-molecule FISH of *Dll1* and *Hes5* expression in spinal cord region corresponding to (B) (Materials and Methods); panels 1 and 2 indicate *Dll1* and *Hes5*, respectively; panels 3 and 4 show false-color merged *Dll1/Hes5* and *Dll1/Hes5/DAPI*, respectively; scale bar 20 μm.

© 2021 The Authors
